# The impact of COVID-19 on life expectancy across socioeconomic groups in Denmark

**DOI:** 10.1186/s12963-024-00323-3

**Published:** 2024-02-07

**Authors:** Cosmo Strozza, Serena Vigezzi, Julia Callaway, José Manuel Aburto

**Affiliations:** 1 Interdisciplinary Centre on Population Dynamics, University of Southern Denmark, Odense, Denmark; 2 Department of Population Health, London School of Hygiene and Tropical Medicine, London, UK; 3 Department of Sociology, University of Oxford, Oxford, UK

**Keywords:** Mortality, Social inequalities, Income, COVID-19 pandemic, Cause of death, Decomposition analysis, Registry data

## Abstract

**Background:**

Denmark was one of the few countries that experienced an increase in life expectancy in 2020, and one of the few to see a decrease in 2021. Because COVID-19 mortality is associated with socioeconomic status (SES), we hypothesize that certain subgroups of the Danish population experienced changes in life expectancy in 2020 and 2021 that differed from the country overall. We aim to quantify life expectancy in Denmark in 2020 and 2021 by SES and compare this to recent trends in life expectancy (2014–2019).

**Methods:**

We used Danish registry data from 2014 to 2021 for all individuals aged 30+. We classified the study population into SES groups using income quartiles and calculated life expectancy at age 30 by year, sex, and SES, and the differences in life expectancy from 2019 to 2020 and 2020 to 2021. We compared these changes to the average 1-year changes from 2014 to 2019 with 95% confidence intervals. Lastly, we decomposed these changes by age and cause of death distinguishing seven causes, including COVID-19, and a residual category.

**Results:**

We observed a mortality gradient in life expectancy changes across SES groups in both pandemic years. Among women, those of higher SES experienced a larger increase in life expectancy in 2020 and a smaller decrease in 2021 compared to those of lower SES. Among men, those of higher SES experienced an increase in life expectancy in both 2020 and 2021, while those of lower SES experienced a decrease in 2021. The impact of COVID-19 mortality on changes in life expectancy in 2020 was counterbalanced by improvements in non-COVID-19 mortality, especially driven by cancer and cardiovascular mortality. However, in 2021, non-COVID-19 mortality contributed negatively even for causes as cardiovascular mortality that has generally a positive impact on life expectancy changes, resulting in declines for most SES groups.

**Conclusions:**

COVID-19 mortality disproportionally affected those of lower SES and exacerbated existing social inequalities in Denmark. We conclude that in health emergencies, particular attention should be paid to those who are least socially advantaged to avoid widening the already existing mortality gap with those of higher SES. This research contributes to the discussion on social inequalities in mortality in high-income countries.

**Supplementary Information:**

The online version contains supplementary material available at 10.1186/s12963-024-00323-3.

## Background

Most high-income countries experienced losses in life expectancy in the first 2 years of the COVID-19 pandemic, with few exceptions. However, in Denmark, life expectancy remained at 2019 levels [1, 2], and in Norway and Finland, life expectancy improved slightly, albeit only among women. It is unknown whether the impact of the pandemic on Danish life expectancy differed by socioeconomic characteristics.

This is important because socioeconomic inequalities in mortality have been widening for both men and women in Denmark, as well as in other Nordic and European countries with high national incomes, social transfers, and healthcare expenditures [3–6]. Moreover, improvements in life expectancy and lifespan inequality (variation in age at death) in high-income countries have differed across socioeconomic strata, with lower socioeconomic groups in Europe and the USA experiencing little improvements by either measure [3, 4, 6–11]. These increasing inequalities in mortality cannot be fully explained by changes in population composition [7, 12].

Socioeconomic inequalities have also been observed in COVID-19 mortality [13, 14]. As a consequence, lower socioeconomic groups, which were already disadvantaged in terms of mortality, have been disproportionately more affected by COVID-19 [15–17]. In the context of Denmark, this raises the following questions: (1) How did different socioeconomic groups experience changes in life expectancy during the COVID-19 pandemic? (2) Did the COVID-19 pandemic exacerbate already existing social inequalities in Denmark? (3) Did the COVID-19 pandemic trigger changes in leading causes of death and their subsequent contributions to life expectancy? In this paper, we address these questions by estimating life expectancy at ages 30 in 2020 and 2021 by socioeconomic status (SES) and comparing this with trends observed from 2014 to 2019. Additionally, we quantify the contributions of age and cause of death, including COVID-19, to expectancy changes.

Although Denmark was relatively unscathed by COVID-19, we hypothesize that socioeconomic groups within the Danish population experienced changes in life expectancy that differed from those of the overall population. Understanding changes in mortality patterns during the pandemic years by SES is an important contribution to existing research on widening socioeconomic inequalities in the Nordic countries. Furthermore, these findings can inform future pandemic, epidemic, or other health policy responses in Denmark by giving nuance to mortality statistics that are not observed at the population level.

## Methods

We used Danish registries from 2014 to 2021 to aggregate data by SES on the entire population aged 30 years or more residing in Denmark on January 1st of each year. Danish registries comprise high quality, individual-level data on the entire resident population of Denmark. The registries include data on income, education, and mortality, which are linked by a unique personal identification number. We used family disposable income as a proxy for SES, which is calculated by adding family members’ individual disposable incomes, including children under the age of 25 living at home. We divided the population into quartiles by family income distribution, computed by year, sex, and 5-year age group, and calculated period life tables by year and sex for each income group separately. From these, we retrieved life expectancy estimates at age 30 by year, sex, and income quartile. To compare results from 2020 and 2021 with those of the previous years, we calculated differences in life expectancy between 2019 and 2020, and 2020 and 2021, and compared them with the average 1-year change between 2014 and 2019 (baseline). We estimated 95% confidence intervals (CIs) by bootstrapping 10,000 populations of 20,000 individuals based on the death distribution of each sub-population’s lifetable to determine statistical significance. Finally, we computed both cause-specific and age- and cause-specific contribution to yearly changes in life expectancy for each sub-population using the linear integral decomposition method [18]. We distinguished eight causes of death: COVID-19, cardiovascular disease (CVD), cancer, suicide and alcohol and drug related (commonly called deaths of despair), accidents (excluding suicide and alcohol and drug related), respiratory diseases, other infectious diseases, and a residual category, which included all other causes of death. The causes of death were all considered to be underlying. This classification of cause of death was used by Aburto et al. [15] when investigating life expectancy losses by race/ethnicity in the USA in 2020 and 2021. It includes some leading causes of death together with other causes that have been discussed during the COVID-19 pandemic. We provide International Classification of Disease-10 codes (ICD-10) for each cause of death in Table 1. We performed a sensitivity analysis using education as proxy of SES. We divided the population into three groups, according to the highest level of educational attainment, following the International Standard Classification of Education: low (less than high school), mid (high school), and high (university diploma).

**Table 1:** Causes of death classified according to ICD-10 codes

Category	ICD-10 codes
COVID-19	U07.1, U07.2
CVD	I00-I78
Cancer	C00-C99
Suicide, alcohol- and drug-related	F10-F16, F19, K70, K73-K74, U03, X40-X45, X64-X85, Y10-Y15, Y87
Accidents	V00-V99, W00-W99, X00-X59, Y85-Y86 (excluding suicide and alcohol- and drug-related deaths)
Respiratory diseases	J40-J46
Other infectious diseases	A00-A09, A16-A44, A48-A99, B00-B09, B15-B99, D86.9, G02, G14, H32, I32, I39 J17, K90.8, L44.4, L94.6, M02.3, M35.2, M66, N34.1, R11.1
Residual	All other causes

*ICD-10* international classification of disease-10, *CVD* cardiovascular disease

## Results

We present results for life expectancy at age 30 by income quartiles in the main text and report results by education as Additional files 1, 2, 3, 4, 5 and 6. There were no qualitative differences between the results.

**Fig. 1:**
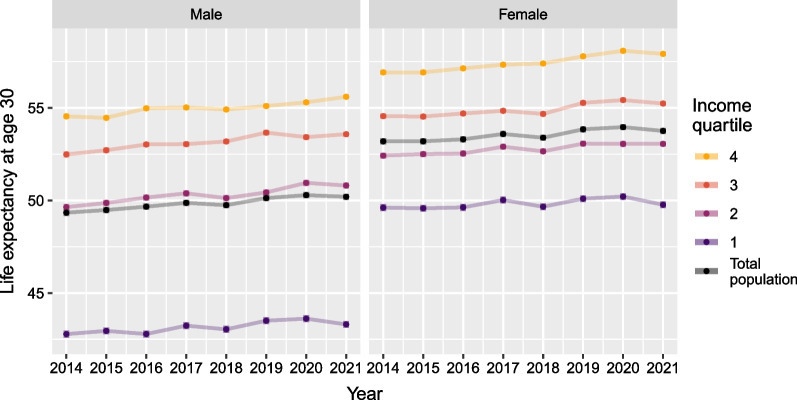
Life expectancy at age 30, with 95% confidence intervals, by sex and income quartile in Denmark. Years 2014–2021

Figure 1 shows life expectancy at age 30, with 95% confidence intervals, by sex and SES for the years 2014 to 2021. Life expectancy increased in the overall population (in black in the figure) from 2014 to 2020, reaching 50.3 years (95% CI 50.1–50.4) for men and 54.0 years (95% CI 53.8–54.1) for women in 2020. When stratifying by SES, we observed a higher remaining life expectancy at age 30 in 2020 among those of higher SES (55.3 years for men, 95% CI 55.2–55.4, and 58.1 years for women, 95% CI 57.9–58.2) than those of lower SES (43.6 for men, 95% CI 43.4–43.8, and 50.2 years for women, 95% CI 50.0–50.4). In 2021, however, we observed a small reduction in life expectancy for both sexes and most of the income groups, except for men in the two highest income quartiles. Figure 1 also shows inequalities in life expectancy improvements over time by SES, with those of higher SES experiencing greater improvements than those of lower SES. Among those of higher SES, life expectancy at age 30 increased from 54.5 years (95% CI 54.4–54.7) in 2014 to 55.6 in 2021 (95% CI 55.5–55.7) for men, and from 56.9 (95% CI 56.8–57.0) to 57.9 (95% CI 57.8–58.0) for women. Among those of lower SES, life expectancy increased 42.8 years (95% CI 42.6–43.0) in 2014 to 43.3 years in 2021 (95% CI 43.1–43.5) for men and from 49.6 years (95% CI 49.4–49.8) to 49.8 years (95% CI 49.6–50.0) for women.

**Fig. 2:**
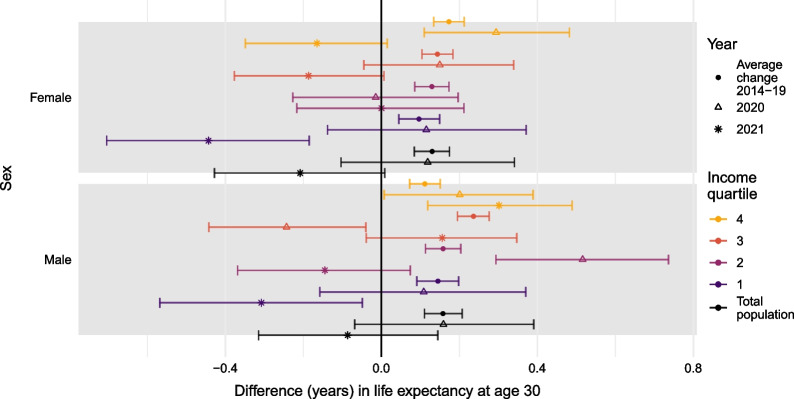
Difference in life expectancy at age 30 by sex and income quartile. Years 2014–2019 (average yearly change), 2019–2020, and 2020–2021

Figure 2 shows the average 1-year change between 2014 and 2019 (circle) compared to yearly differences in life expectancy from 2020 to 2019 (triangle) and 2021 to 2020 (asterisk). This gives further insight into changes in life expectancy during the pandemic years and contextualizes them with pre-pandemic results. The estimates are presented with 95% confidence intervals.

As illustrated in Fig. 2, total male and female life expectancies increased during the pre-pandemic years (2014 to 2019) and from 2019 to 2020, but decreased from 2020 to 2021, although none of the changes were statistically significant during the pandemic. The same pattern was observed among women in all income quartiles except the second, who did not experience any change throughout the pandemic period. The increase in life expectancy in 2020 for those in the fourth income quartile and the decrease in 2021 for the first income quartiles represented statistically significant changes. Increases in life expectancy in 2020 and decreases in 2021 followed the social gradient; those of higher SES experienced greater improvement (2020) and slower decrease (2021) compared to those of lower SES. Changes in life expectancy among men in the first and second income quartiles followed those of the total population. Men in the third income quartile experienced a reduction in life expectancy in 2020, followed by a stark increase in 2021, with the first being statistically significant. Finally, men in the fourth income quartile experienced an improvement throughout the two pandemic years, which in 2021 was statistically significantly higher than changes observed among men in the lowest income quartile. In 2021, the pattern of changes in life expectancy across all income groups resembles the social gradient in mortality, suggesting that COVID-19 increased inequalities in life expectancy across income groups for men as well as women.

**Fig. 3:**
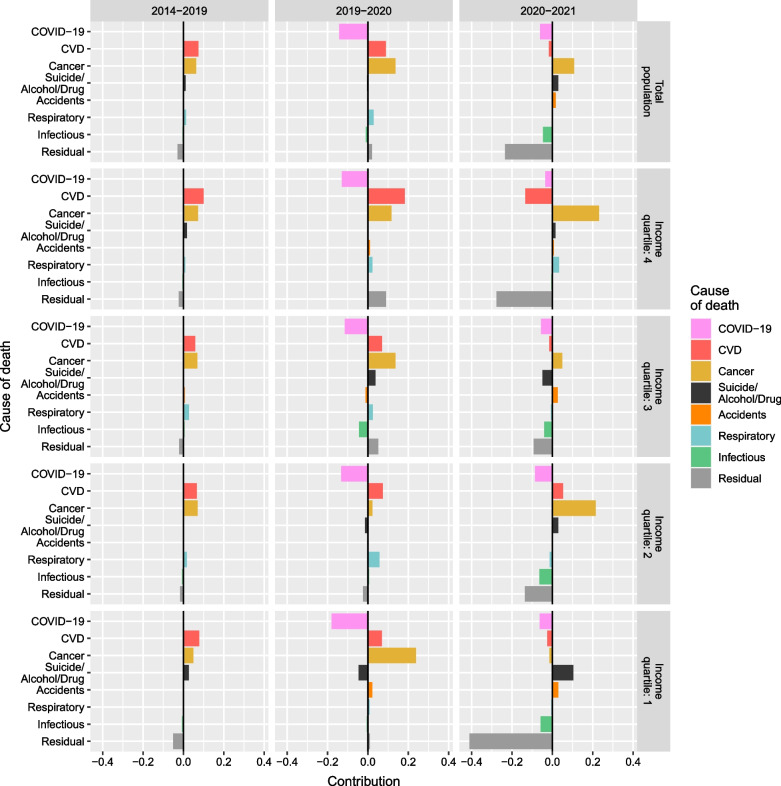
Cause-specific contribution to changes in life expectancy at age 30 by income quartile among women. Years 2014–2019 (average), 2019–2020, and 2020–2021

**Fig. 4:**
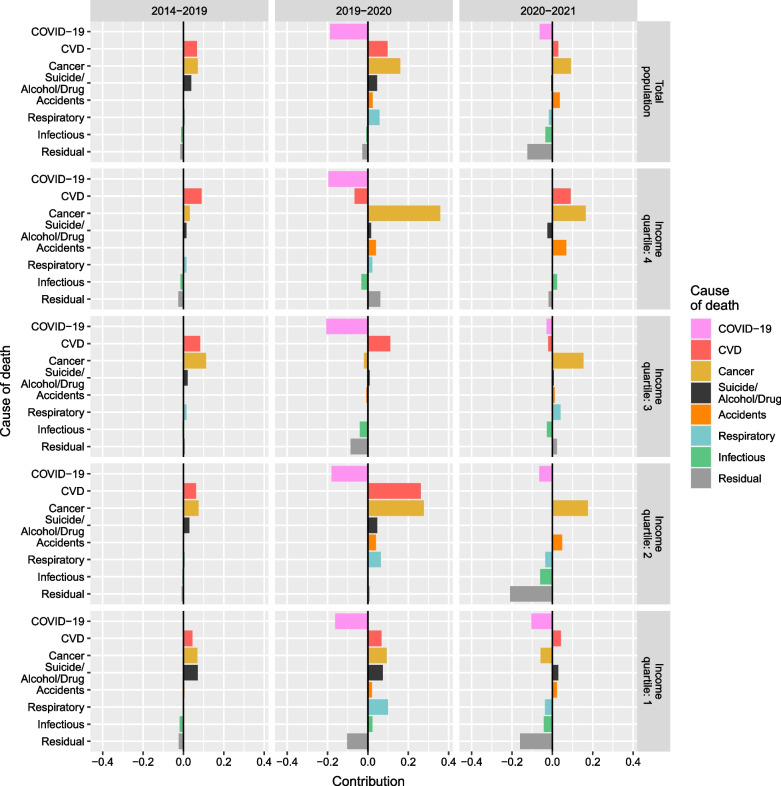
Cause-specific contribution to changes in life expectancy at age 30 by income quartile among men. Years 2014–2019 (average), 2019–2020, and 2020–2021

Figures 3 and 4 depict results of the decomposition analysis by cause of death, by SES for women and men, respectively. For both, most gains in life expectancy were driven by improvements in cancer and CVD mortality prior to the pandemic. This trend was observed for the total population as well as across income groups. While it continued during the pandemic years, with some variation across income groups, COVID-19 contributed negatively to changes in life expectancy in both 2020 and, to a lesser degree, 2021. During the pandemic, the contributions of other causes also changed. In 2020, some causes that did not substantially affect life expectancy before the pandemic contributed positively (e.g., respiratory diseases or suicide and alcohol- and drug-related deaths among men). In contrast, other causes started contributing negatively in 2021 (e.g., other infectious diseases and the residual category).

**Fig. 5:**
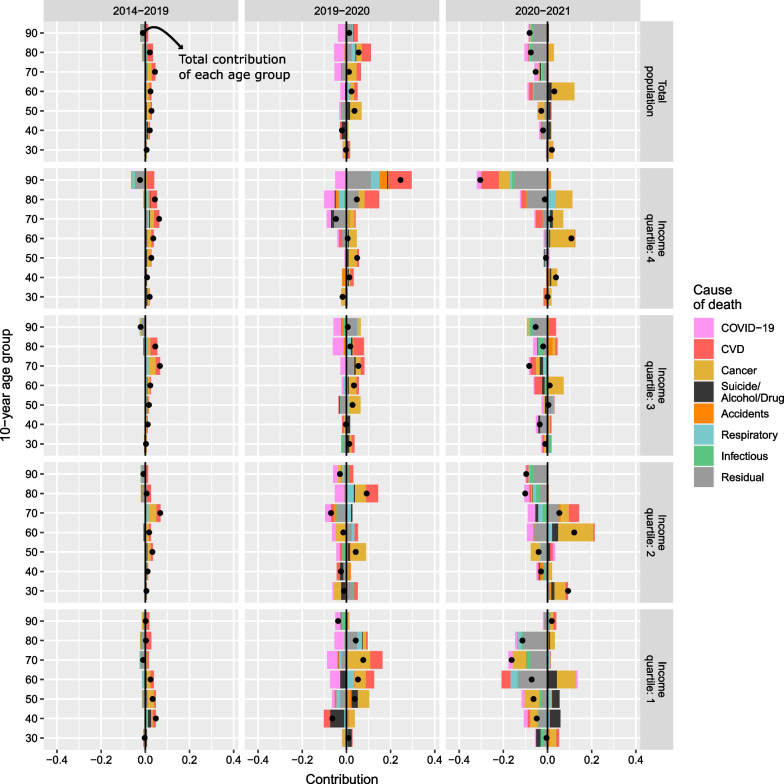
Age- and cause-specific contribution to changes in life expectancy at age 30 by income quartile among women. Years 2014–2019 (average), 2019–2020, and 2020–2021

**Fig. 6:**
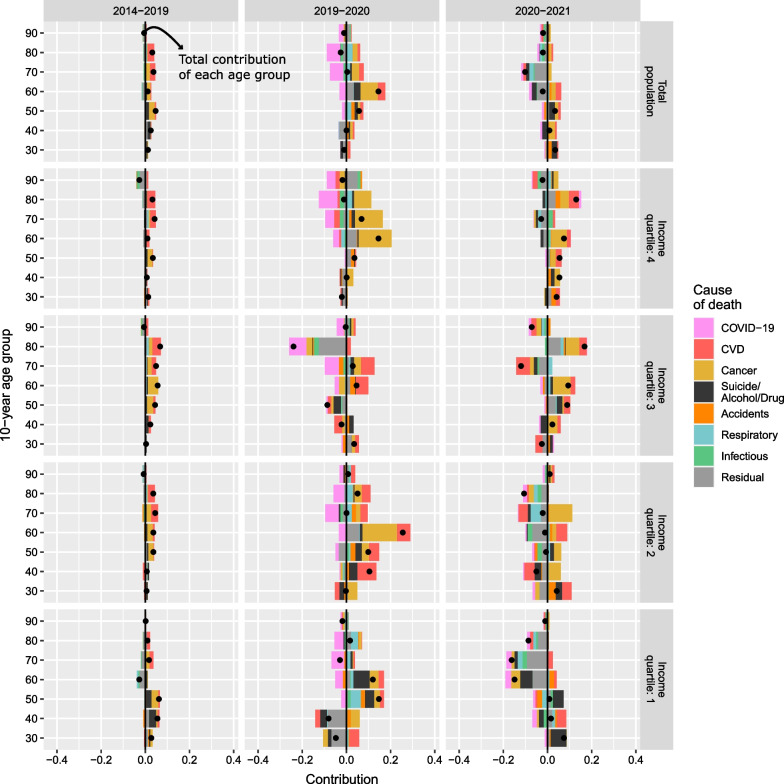
Age- and cause-specific contribution to changes in life expectancy at age 30 by income quartile among men. Years 2014–2019 (average), 2019–2020, and 2020–2021

Figures 5 and 6 depict results of the decomposition analysis by age group and cause of death of female and male life expectancy differences across income groups, respectively. For the total female and male populations, changes in 2020 were driven negatively by COVID-19 mortality for ages 50+. These were counterbalanced by larger positive contributions from changes in non-COVID-19 mortality, predominantly cancer mortality, which were observed in both sexes at the same ages.

For women, this positive contribution existed during the 5 years prior to the pandemic at all ages, peaking at ages 70–79, with the only negative contribution of mortality being from residual causes observed after age 80. In 2021, both non-COVID-19 and COVID-19 mortality contributed negatively to changes in life expectancy, with the main driver of life expectancy losses attributed to other infectious diseases and residual causes after age 80. Cancer mortality improved, partially offsetting the negative impact of COVID-19. Results for women in the first, third, and fourth income quartiles reflected similar patterns as those for the total female population. Women in the lowest income group experienced greater decreases and smaller increases in life expectancy than women in the higher groups. This was driven by differences in suicide and alcohol- and drug-related death and respiratory diseases in 2020, and mainly cancer, respiratory diseases, and residual causes in 2021.

Women in the second income group showed no coherent patterns in age- and cause-specific contributions in 2020 or 2021. In 2020, negative contributions from suicide and alcohol- and drug-related death, CVD, and residual causes at ages 40–49 and accidents, CVD, and residual causes at ages 70–79 counterbalanced the positive effect of other causes at ages 50–59 (mainly cancer), and 80–89 (mainly accidents, cancer, and CVD). In 2021, a negative mortality contribution was observed, especially at the oldest ages. This was counterbalanced by an improvement in mortality at the ages in which mortality had worsened in 2020. In both years, COVID-19-mortality negatively contributed to changes in life expectancy, but contributions were greater in 2020 than in 2021.

Among men, adult and old age mortality improved in the pre-pandemic years, and only worsened from age 90 onwards. In 2020, life expectancy generally increased across income groups because the positive contributions from non-COVID-19 mortality, greatly increased in both magnitude and number of causes from pre-pandemic trends, counterbalanced the negative contributions from COVID-19 mortality.

The main exception to the general positive trend is men in the third income quartile, for whom life expectancy did not increase in 2020. In this case, changes were driven by negative contributions of CVD, suicide and alcohol- and drug-related death at younger adult ages (40–59), and cancer, other infectious diseases, and residual causes at older ages (80–89). Men in the fourth income quartile experienced negative contributions caused by an increase in CVD mortality after age 60 that were, however, counterbalanced by the positive contributions of other causes (largely cancer).

In 2021, both non-COVID-19 and COVID-19 mortality contributed negatively to life expectancy changes, with some positive contributions from changes in non-COVID-19 mortality at younger adult ages (30–39, 50–59) mainly by cancer and CVD, but also suicide and alcohol- and drug-related death, and accidents. The first two income groups saw a reduction in life expectancy, driven, generally, by negative contributions of both COVID-19 and non-COVID-19 mortality, predominantly by respiratory diseases, suicide and alcohol- and drug-related death, and residual causes at ages above 60. Additionally, few to no improvements in cancer mortality were observed. For those in the third and fourth income quartiles, life expectancy increased because of moderate improvements in non-COVID-19 mortality at most ages, with cancer, CVD, respiratory diseases, and other infectious diseases positively contributing to changes in life expectancy, as well as very low COVID-19 mortality.

## Discussion

In contrast with nearly all high-income countries, Denmark did not see a decrease in life expectancy in 2020 [1]. COVID-19 mortality did contribute negatively to changes in life expectancy, but Denmark’s COVID-19 policy interventions, which included mask mandates and lockdowns, in conjunction with its strong healthcare system and public trust in institutions, were considered successful in mitigating the effects of the pandemic [19]. The negative effects of COVID-19 mortality on life expectancy were totally countered by positive contributions of non-COVID-19 mortality at adult and older ages, particularly because of improvements in cancer and cardiovascular mortality. In 2021, when many countries experienced an increase in life expectancy, Denmark experienced a small loss, declining to the levels seen before the pandemic, in 2019. Denmark’s COVID-19 vaccination program resulted in a rate of over 80% vaccinated with at least one dose by the end of 2021, which diminished the effects of the COVID-19 pandemic on changes in mortality [2]. This played a role in limiting the impact of COVID-19 mortality on changes in life expectancy. However, mortality by other causes did contribute to life expectancy losses, especially at older ages.

One of our research questions pertains to the effects of the pandemic on the non-COVID mortality contributions to life expectancy changes. The literature confirms our finding that over the past decades, CVD mortality has contributed positively to changes in life expectancy [20, 21]; however, we also found that the effect reversed for some groups between 2020 and 2021. This is consistent with Aburto et al. [15], who find an increase in the contribution of unintentional injuries and a reversal of the contribution of CVD mortality to life expectancy changes in the USA during the pandemic.

In the early stages of the COVID-19 pandemic, Marmot and Allen [22] anticipated that it would expose and amplify inequalities in societies. Such phenomena would be observed through different exposures to the virus, for example, the possibility of working from home and ability to adhere to measures imposed to limit the spread of the virus. Throughout the COVID-19 pandemic, studies observed a social gradient in COVID-19 mortality, with those of lower SES experiencing higher mortality than those of higher SES [13, 14]. This was observed in different high-income settings and across a range of SES-associated indicators, including income, education level, unemployment, poverty level, household crowding, and housing conditions [16, 23–27]. Given the known socioeconomic inequalities in mortality in Denmark [6, 12, 28], we hypothesized that mortality patterns during the COVID-19 pandemic would differ across the socioeconomic sphere when compared to the national average. We ask, has COVID-19 exacerbated already existing inequalities in mortality across income groups?

To answer this question, we focused on life expectancy as a measure of population health, comparable across countries and time. Life expectancy losses do not represent the number of years that will be deducted from the lifespans of individuals who were living during the pandemic years; rather, it quantifies the impact of a mortality shock generated in a specific period [29]. This study represents a novel contribution to the literature on the impact of the COVID-19 pandemic on life expectancy changes using individual-level data. By utilizing the Danish population registry, we were able to evaluate the impact of the pandemic on the social inequalities in mortality characterizing the population.

We analyzed yearly life expectancy changes in the period prior to the pandemic (2014–2019 average yearly changes) and compared them to changes that occurred during the pandemic years (2019–2020 and 2020–2021) by sex and income group. Among women, we observed the well-established social gradient in mortality in both 2020 and 2021; life expectancy increased more in 2020 and decreased less in 2021 among those with higher incomes compared to those with lower incomes. COVID-19 mortality had a larger negative impact on life expectancy among those with lower income. This could be tied to occupational differences among women in the lower-income groups that might have put them at higher risk of COVID-19 infection. At the same time, the contribution of non-COVID-19 mortality has been crucial in determining the difference in life expectancy losses across income groups. For instance, while negative contributions to life expectancy before the pandemic were restricted to ages above 70, in 2021 the lowest income group experienced high negative contributions from non-COVID-19 mortality, mainly attributable to cancer, respiratory diseases, and residual causes, from age 40 onwards.

We observed very different results in the male population. There was a general improvement in life expectancy in 2020. This could be because men were mitigated from the riskier health behaviors leading to large positive contributions of non-COVID-19 mortality to life expectancy changes. We observed a reduction in mortality attributable to accidents, mainly at ages 30–69. Generally, men exhibit more risky health behaviors than women, for example, in alcohol consumption [30, 31], and smoking [32]. They also access healthcare less frequently and later than women [33]. Previous research also indicates that those who experience traffic injuries in Denmark are predominantly male [34], and Trier et al. [35] observed a reduction in traffic-related injuries at a major trauma center in Denmark during the lockdown, which could have affected male mortality more than female mortality during this time. The age-specific non-COVID-19 mortality contributions generally resembled pre-pandemic patterns, with some exceptions. For the lowest income group, contrary to what we observed in the pre-pandemic years, contributions were negative at ages 30–49, driven by residual causes, and positive at ages 60–69, attributable to cancer and suicide and alcohol- and drug-related death.

In 2021, the picture changed, and those with higher incomes experienced an increase in life expectancy, while those with low income experienced a decrease. COVID-19 mortality had a greater negative contribution among the lower income groups, while it was nearly absent in the highest. Despite universal healthcare coverage, socioeconomic differences in COVID-19 vaccine uptake were observed in Denmark. In a registry-based study of COVID-19 vaccination coverage in Denmark, Gram et al. [36] found that among those with the highest odds of non-vaccination were descendants of non-Western immigrants and those with primary school as their highest completed level of education—two indicators of lower SES in Denmark—but also those with high disposable incomes—an indicator of high SES. However, those of high SES in Denmark were most likely protected against increased levels of COVID-19 mortality due to other social factors. At the same time, the main driver of the difference across income groups has been non-COVID-19 mortality, with large negative contributions at all adult and older ages among those with the lowest income. This is attributable mainly to mortality respiratory diseases, suicide and alcohol- and drug-related death, and residual causes. In general, as with women, but more dramatically, COVID-19 enlarged the gap between income groups in terms of life expectancy.

Most of our results depict the social gradient in mortality during the COVID-19 pandemic. It confirms our hypothesis that COVID-19 exacerbated already existing inequalities in mortality across income groups, as observed in other high-income countries [17, 37, 38]. However, we also observe some results that fall out of this pattern. For instance, women in the second income group did not experience any change in life expectancy in either 2020 or 2021. Their higher mortality, compared to the other income groups, in 2020 was counterbalanced by lower mortality in 2021. Age-specific contributions of mortality from causes other than COVID-19 deviated from the pre-pandemic patterns in 2020 but mostly bounced back in 2021. This could be because of the occupations that women in this income group have, with a higher share of essential workers than in the other groups, who were at higher risk of mortality in 2020 but lower in 2021 due to early vaccinations [36, 39]. Furthermore, men in the third income quartile experienced a large decrease in life expectancy when compared to pre-pandemic levels in 2020, followed by an increase in 2021. While this could be due to yearly fluctuation in the data, we observed the biggest contributions to change in mortality at ages 50–59 and 80–89. Looking at ages 50–59, the explanation for this result could be related to the occupations performed by those in this income group. For ages 80–89, it is likely associated with the causes of death profile of this income group at this period in time.

Additionally, men in the highest two income groups were the only ones to see an increase in life expectancy from 2020 to 2021. It is well established that having a higher SES is a protective factor against mortality caused by COVID-19 and other infectious diseases due to social factors, such as higher education levels and better access to healthcare [13, 40, 41]. However, it is also possible that men in higher-income groups predominantly have occupations that were performed from home during the COVID-19 lockdown, and thus, were not as at high risk of COVID-19 infection. These results lay the groundwork for further research into socioeconomic differences in occupational mortality during COVID-19.

This study has limitations that need to be acknowledged. Although the study is based on data from the population registry, the relatively small Danish population makes year-by-year changes and the age-cause mortality decomposition more volatile. Death counts by sex, age, income, and cause might be very small, or even null, for a single year. This explains the relatively large confidence intervals in year-to-year changes. Furthermore, the ability to distinguish individuals by income group becomes less efficient at older ages, where income inequalities tend to diminish. For this reason, we performed a sensitivity analysis by education, which confirmed the general trend observed in our results.

## Conclusions

To conclude, inequalities across income groups increased in Denmark for both women and men. Among women in 2021, life expectancy decreased for each group, but to a larger extent for those with lower incomes. Among men, there was a clear distinction between higher- and lower-income groups, with life expectancy increasing in the former and decreasing in the latter. These inequalities in mortality were exacerbated by the direct and indirect effect of COVID-19, which disproportionately affected those of lower SES. Our results suggest that in health emergencies, particular attention should be paid to those who are most socially disadvantaged to avoid increasing an already existing survival gap with the rest of the population.

### Supplementary Information

**Additional file 1**. **Figure S1**. Life expectancy at age 30, with 95% confidence intervals, by sex and education in Denmark. Years 2014–2021.

**Additional file 2**. **Figure S2**. Difference in life expectancy at age 30 by sex and education. Years 2014–2019 (average yearly change), 2019–2020, and 2020–2021.

**Additional file 3**. **Figure S3**. Cause-specific contribution to changes in life expectancy at age 30 by education among women. Years 2014–2019 (average), 2019–2020, and 2020–2021.

**Additional file 4**. **Figure S4**. Cause-specific contribution to changes in life expectancy at age 30 by education among men. Years 2014–2019 (average), 2019–2020, and 2020–2021.

**Additional file 5**. **Figure S5**. Age- and cause-specific contribution to changes in life expectancy at age 30 by education among women. Years 2014–2019 (average), 2019–2020, and 2020–2021.

**Additional file 6**. **Figure S6**. Age- and cause-specific contribution to changes in life expectancy at age 30 by education among men. Years 2014–2019 (average), 2019–2020, and 2020–2021.

## Data Availability

Access to individual-level data is restricted by Danish law. Danish registry data can be accessed with authorization by Statistics Denmark. R code is available upon request.

## Abbreviations

CVD: Cardiovascular disease
SES: Socioeconomic status
