# Supplementary figures and images for: The impact of COVID-19 on life expectancy across socioeconomic groups in Denmark
Source: Popul Health Metr. 2024 Feb 7;22:3. doi: 10.1186/s12963-024-00323-3 (PMC10848407; doi:10.1186/s12963-024-00323-3)

Life expectancy at age 30

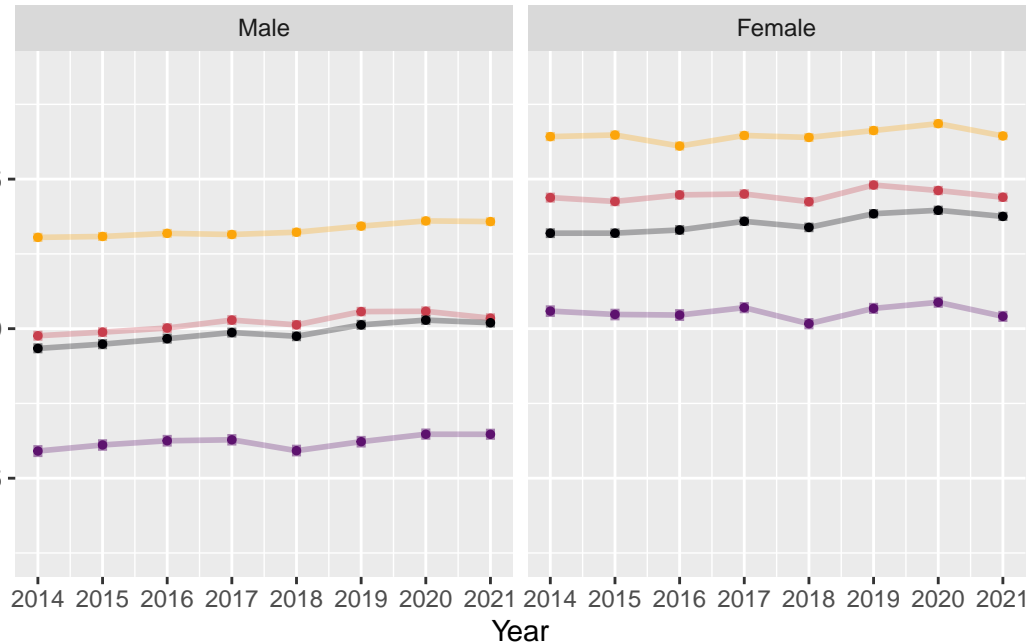

Supplement: Supplementary file 1 — Additional file 1. Figure S1. Life expectancy at age 30, with 95% confidence intervals, by sex and education in Denmark. Years 2014–2021. [file 12963_2024_323_MOESM1_ESM.pdf]

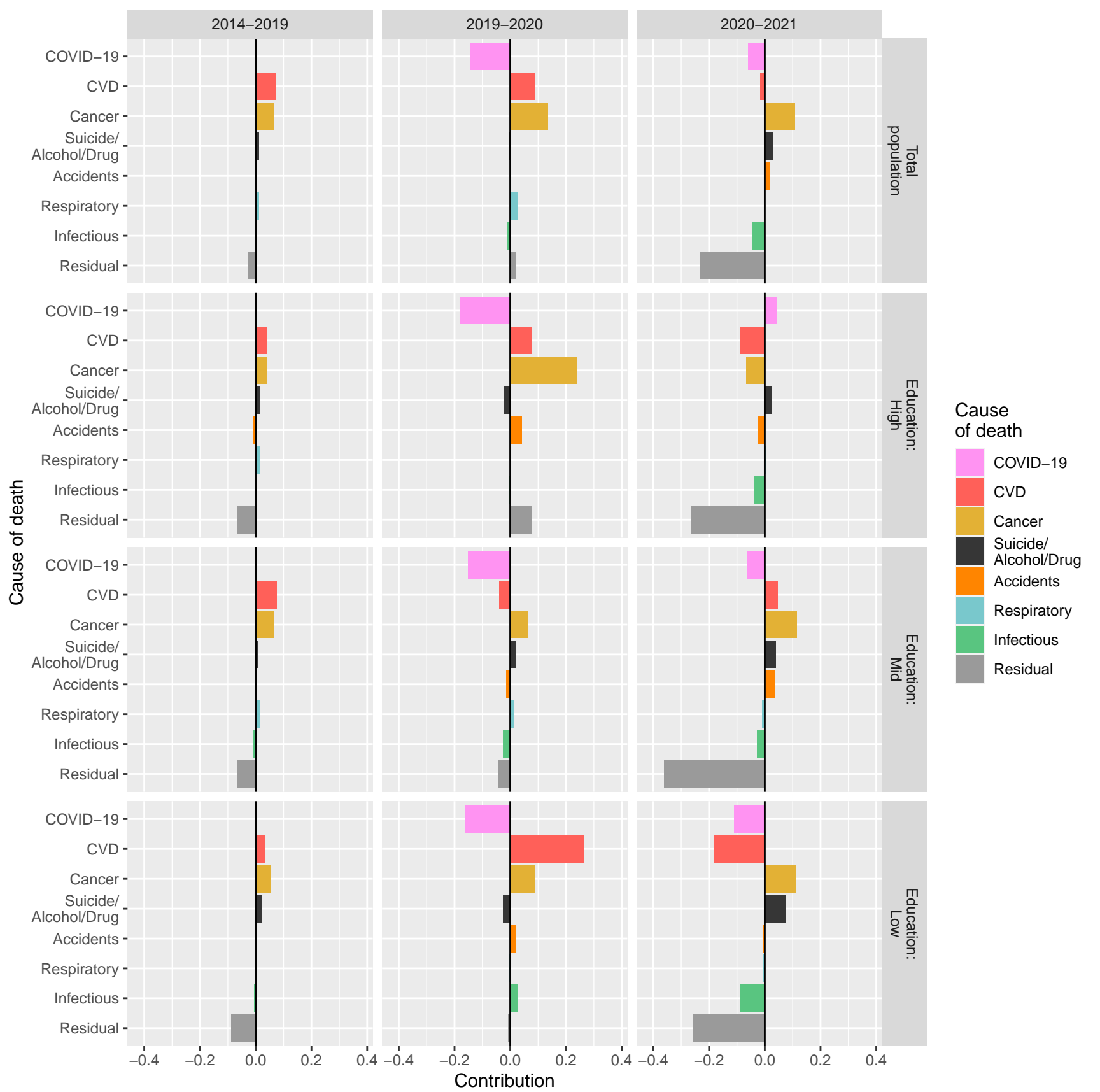

Supplement: Supplementary file 3 — Additional file 3. Figure S3. Cause-specific contribution to changes in life expectancy at age 30 by education among women. Years 2014–2019 (average), 2019–2020, and 2020–2021. [file 12963_2024_323_MOESM3_ESM.pdf]

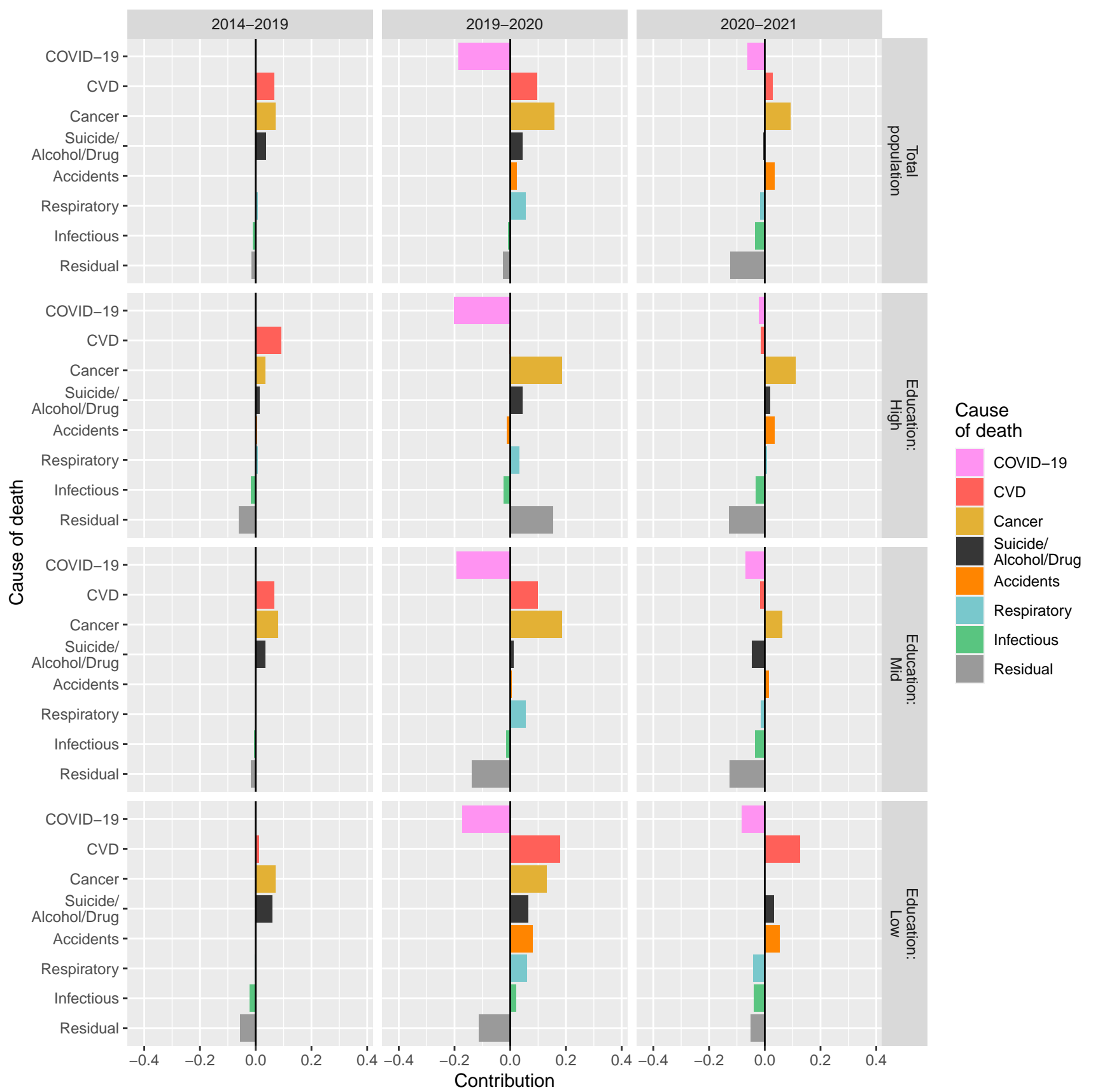

Supplement: Supplementary file 4 — Additional file 4. Figure S4. Cause-specific contribution to changes in life expectancy at age 30 by education among men. Years 2014–2019 (average), 2019–2020, and 2020–2021. [file 12963_2024_323_MOESM4_ESM.pdf]

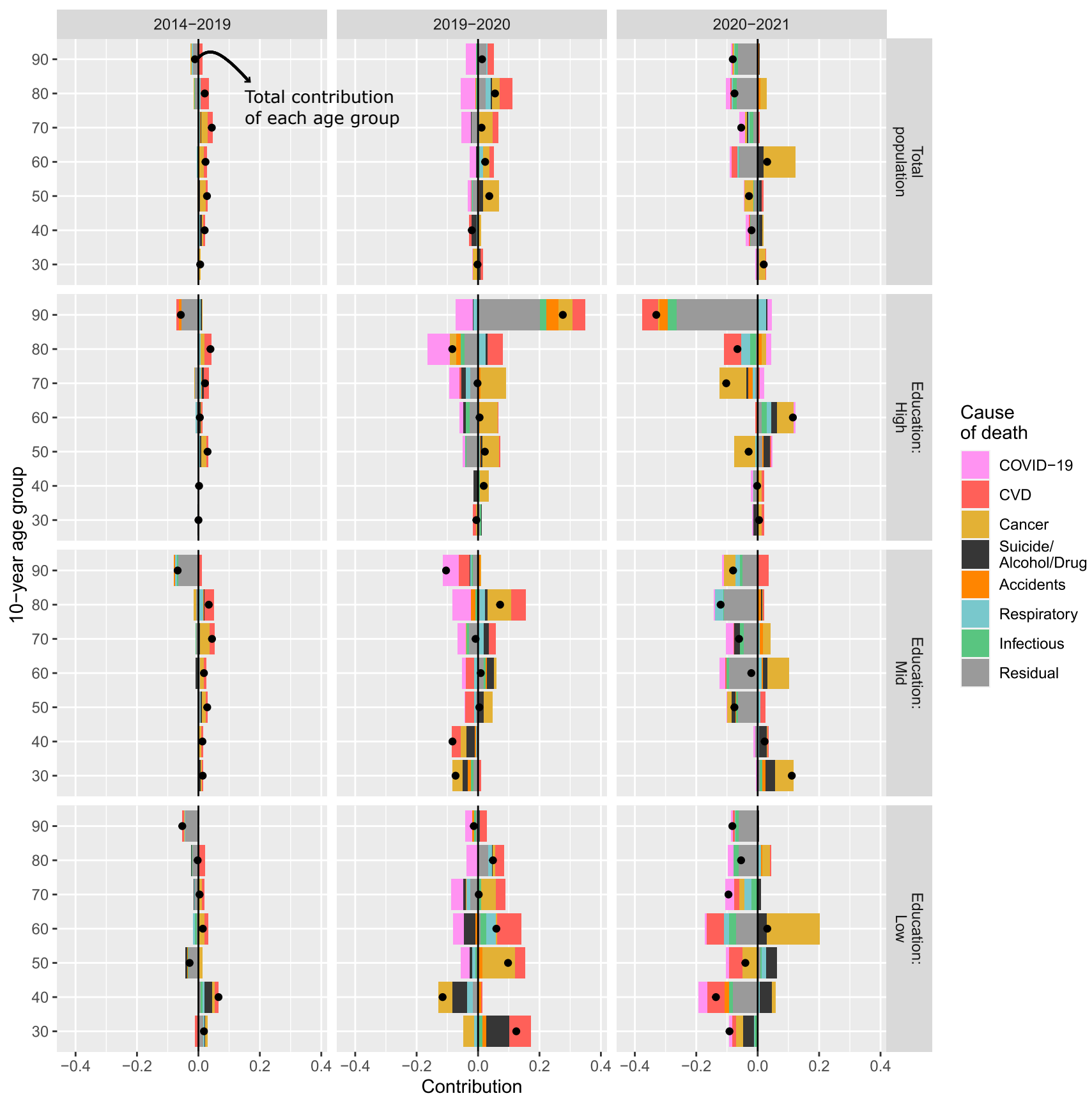

Supplement: Supplementary file 5 — Additional file 5. Figure S5. Age- and cause-specific contribution to changes in life expectancy at age 30 by education among women. Years 2014–2019 (average), 2019–2020, and 2020–2021. [file 12963_2024_323_MOESM5_ESM.pdf]

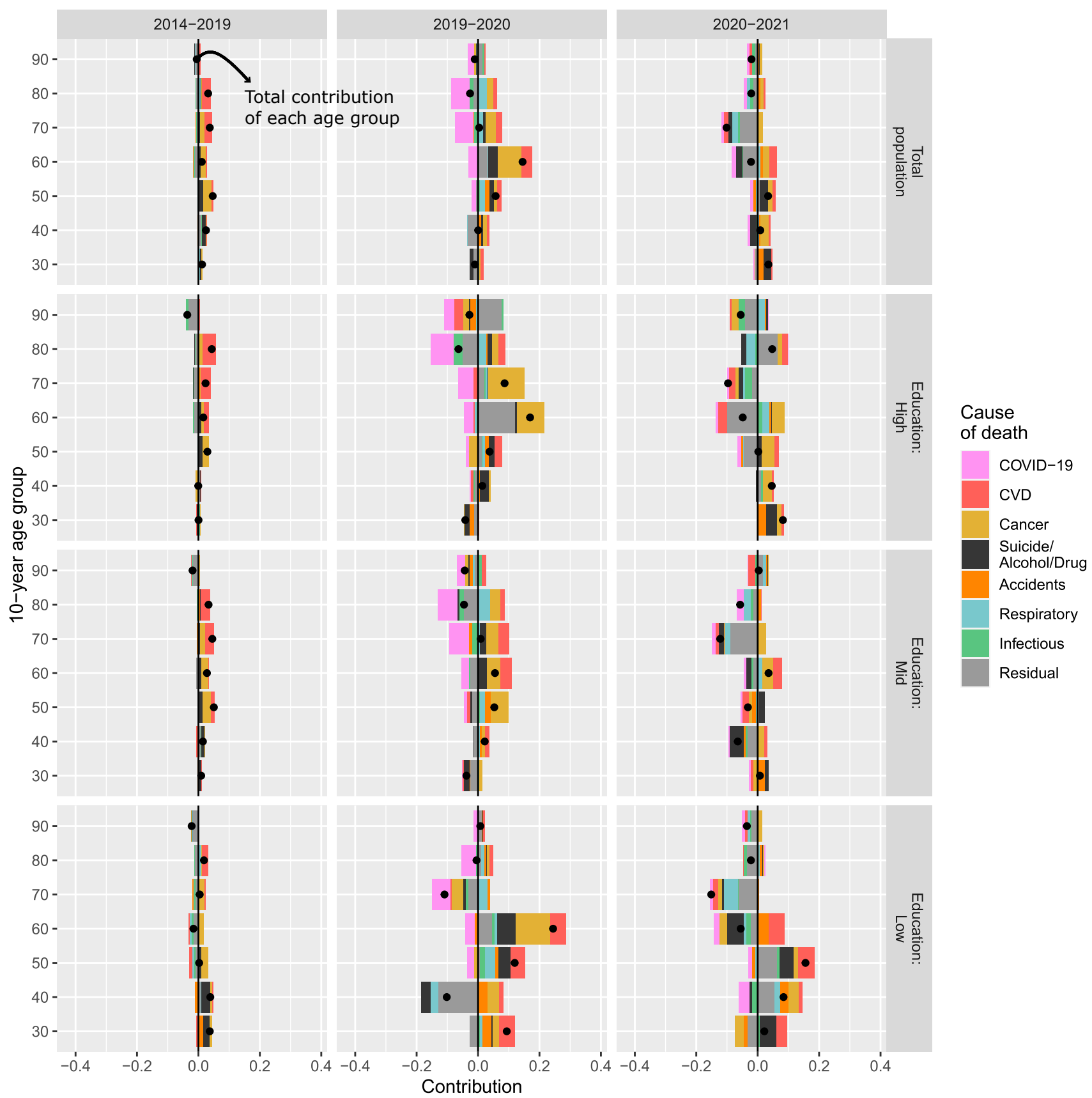

Supplement: Supplementary file 6 — Additional file 6. Figure S6. Age- and cause-specific contribution to changes in life expectancy at age 30 by education among men. Years 2014–2019 (average), 2019–2020, and 2020–2021. [file 12963_2024_323_MOESM6_ESM.pdf]
