# Supplementary material for: The impact of COVID-19 on life expectancy across socioeconomic groups in Denmark
Source: Popul Health Metr. 2024 Feb 7;22:3. doi: 10.1186/s12963-024-00323-3 (PMC10848407; doi:10.1186/s12963-024-00323-3)
Supplement: Supplementary file 2 — Additional file 2. Figure S2. Difference in life expectancy at age 30 by sex and education. Years 2014–2019 (average yearly change), 2019–2020, and 2020–2021. [file 12963_2024_323_MOESM2_ESM.pdf]

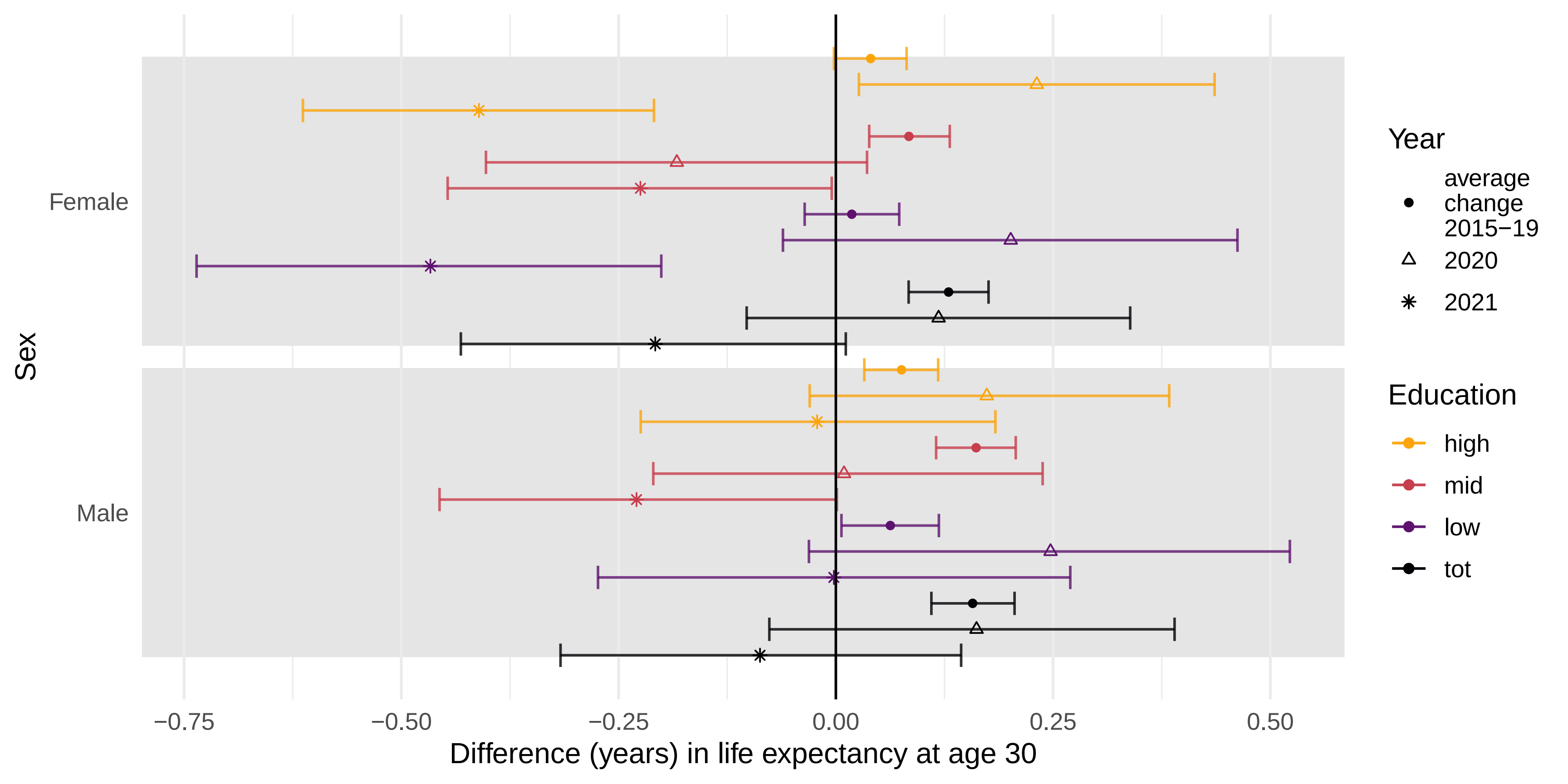

Female
Male
Sex
−0.75
−0.50
−0.25
0.00
0.25
0.50
Difference (years) in life expectancy at age 30
Year
average change 2015−19
2020
2021
Education
high
mid
low
tot
